# Modulation Technique of Localized Surface Plasmon Resonance of Palladium Nanospheres by Coating with Titanium Dioxide Shell for Application to Photothermal Therapy Agent

**DOI:** 10.1186/s11671-022-03697-1

**Published:** 2022-06-23

**Authors:** Yutaro Hayakawa, Masato Furuya, Hironobu Tahara, Yasuhiro Kosuge, Tsuyoshi Kimura, Kosuke Sugawa, Joe Otsuki

**Affiliations:** 1 Department of Materials and Applied Chemistry, College of Science and Technology, Nihon University, Chiyoda, Tokyo 101-8308 Japan; 2 Graduate School of Engineering, Nagasaki University, Bunkyo, Nagasaki, 852-8521 Japan; 3 Laboratory of Pharmacology, School of Pharmacy, Nihon University, 7-7-1 Narashinodai, Chiba, Funabashi 274-8555 Japan; 4 Institute of Biomaterials and Bioengineering, Tokyo Medical and Dental University, Chiyoda, Tokyo 101-0062 Japan

**Keywords:** Localized surface plasmon resonance, Palladium nanospheres, Photothermal therapy, Photothermal conversion, Titanium dioxide

## Abstract

Although plasmonic palladium (Pd) nanospheres are thermodynamically stable and have high photothermal conversion due to the free and bound electron coupling associated with the intrinsic high interband transition, they have not attracted attention as a photothermal conversion material for next-generation photothermal cancer therapy. This is because the Pd nanospheres generate the localized surface plasmon resonance (LSPR) intrinsically in the ultraviolet region, which is far away from the biological transparent window (750–900 nm). In this study, we controlled the LSP wavelength of Pd nanospheres by coating with high refractive index TiO_2_ shells taking advantage of the Pd LSPR which is highly sensitive to changes in the local refractive index around the nanospheres. Our calculations indicated that the absorption cross section at 808 nm (corresponding to the wavelength used for photothermal treatment) was increased by 4.5 times by redshifting the LSPR and increasing the extinction intensity associated with the coating with TiO_2_ shell. Experiments confirmed the theoretical prediction in that the LSPR of the synthesized Pd nanospheres with a diameter of 81 nm was significantly redshifted by coating with amorphous TiO_2_ shell, resulting in significant large extinction intensity at 808 nm. The photothermal conversion efficiency was estimated to be 50%. In vitro cell tests, HeLa cells incubated with 100–300 μg/mL TiO_2_-coated Pd nanospheres were efficiently killed by irradiating 808 nm laser (1.8 W) even though the nanospheres with the same concentrations showed little cytotoxicity. These results indicate that the Pd nanospheres coated with high refractive index shells can be promising as a photothermal therapy agent.

**Supplementary Information:**

The online version contains supplementary material available at 10.1186/s11671-022-03697-1.

## Introduction

Localized surface plasmon resonance (LSPR) of metal nanoparticles has been one of the most important optical phenomena for the development of photothermal therapy techniques as a next-generation noninvasive cancer therapy [1–5]. To maximize the useful effects of LSPR in the technology, to control the wavelength and intensity of LSPR is important. The LSPR of metal nanoparticles incorporated near the tumor region is excited by irradiating laser light in the near-infrared region (biological transparent window: 750–900 nm) where the penetration depth into bio-tissues is large [6]. The strong local heat generated during the deactivation of the LSPR leads to the death of cancer cells. Therefore, the metal nanoparticles of which a strong LSPR can be generated in the biological transparent window must be developed. Although the gold (Au) nanoparticles with high chemical stability in biological environments are considered one of the most useful plasmonic photothermal therapy agents, Au nanospheres generally show an LSPR at approximately 500 nm, which is much shorter than the biological transparent window. To tune the LSPR of Au toward the biological transparent window, anisotropic Au nanoparticles have been developed intensely, including Au nanorods [5], Au nanostars [7], Au nanoprisms [8], and so on. However, the morphology of these anisotropic nanoparticles is intrinsically unstable under photothermal conditions in that their apexes (protrusions) are vulnerable to melting. In addition, cetyltrimethylammonium bromide (CTAB), which is often required as a protective agent to realize the morphological anisotropy [9–12], has a high cytotoxicity [13, 14]; complete removal of the surfactant is still difficult [15–21]. In short, an alternative approach is desired for tuning the LSPR wavelength to the biological transparent window.

Palladium (Pd) nanoparticles are one of the plasmonic metal nanomaterials having a high imaginary part of dielectric function derived from the intrinsic interband transition across a wide UV–visible-near-infrared region [22, 23]. Therefore, the Pd nanoparticles may have a high photothermal conversion efficiency through the enhanced formation of excited electron–hole pairs by the coupling between the free- and bound-electron response of palladium [24]. In addition, Pd nanoparticles have an intrinsically high chemical stability in the biological environments. However, the usefulness of Pd nanospheres as a photothermal therapeutic material is considered to be limited since they generate the LSPR only in the UV region. Consequently, the application of Pd LSPR to photothermal conversion is limited to only a few examples [25–27], while attention to anisotropic Pd nanoparticles has been increasing recently. We recently found that the LSPR wavelength of Pd nanospheres is more sensitive to a change in the refractive index of their surrounding environment, as compared with Au and Ag nanospheres [28]. This is attributed to the small wavelength dispersion of real part of the Pd dielectric function. This finding prompted us to consider an expectation that the LSPR wavelength of Pd nanospheres could be significantly redshifted by hybridization with a high refractive index material. In this study, the useful optical specificity as a photothermal therapy agent of the Pd nanosphere, which is coated with a high refractive index semiconductor, was theoretically demonstrated through the comparison with a bare Pd nanosphere and general Au nanosphere coated with the semiconductor. Our experiments corroborated the theoretical calculations; the LSPR wavelength of synthesized Pd nanospheres was significantly redshifted by coating with a titanium dioxide (TiO_2_) shell, resulting in a significant increase in the extinction intensity at 808 nm, which corresponds to the laser wavelength used for the photothermal therapeutic treatment. It has been further demonstrated in this study that the Pd nanospheres coated with TiO_2_ shells show a low cytotoxicity and efficient photothermal therapeutic performance in vitro. There are only a few reports that employ the strategy of coating a plasmonic core with a high refractive index shell to shift the LSP resonance to the longer wavelength for the development of effective photothermal therapeutic nanomaterials [29, 30]. Although anisotropic Au nanoparticles (Au nanorods and nanoplates) used as a core have higher refractive index sensitivities than Au nanospheres and exhibit LSP resonance in the near-infrared region, highly cytotoxic CTAB was used for the anisotropic growth of Au cores. Therefore, a major challenge in this study is to develop effective photothermal therapeutic materials by using spherical plasmonic metal nanoparticles as a core, which do not require CTAB for the synthesis, and coating with a high refractive index shell.

## Methods

### Materials

Deionized water (resistivity: 18.2 MΩ cm^−1^), which was obtained from a Milli-Q water purification system, was utilized for the preparation of all aqueous solutions. Hydrogen tetrachloroaurate(III) tetrahydrate (HAuCl_4_·4H_2_O, Nacalai Tesque), trisodium citrate dihydrate (Kanto Chemical, Japan), L-ascorbic acid (Fujifilm Wako Pure Chemical, Japan), palladium(II) chloride (PdCl_2_, Fujifilm Wako Pure Chemical, Japan), hydrochloric acid (HCl, Fujifilm Wako Pure Chemical, Japan), hydroxypropyl cellulose (Sigma-Aldrich, USA), 2-propanol (Kishida Chemical, Japan), ammonium aqueous solution (28 wt%, Kishida Chemical, Japan), titanium diisopropoxide bis(acetylacetonate) (TDAA, Sigma-Aldrich, USA), and 8-ArmPEG-Amine (20,000 Da, Biopharma PEG Scientific, USA) were used as received. Dulbecco's Modified Eagle's Medium (D-MEM, low glucose) with L-glutamine and phenol red and PBS(-) were obtained from Fujifilm Wako Pure Chemical, Japan. Penicillin streptomycin, trypsin–EDTA (0.25%), and fetal bovine serum (FBS) were obtained from Gibco, USA. HeLa cell lines were purchased from JCRB Cell Bank, Japan.

### Synthesis of Au(core)/Pd(shell) Nanoparticles (Au/PdNSs, Step 1 and 2 in Scheme 1).

**Scheme 1:**
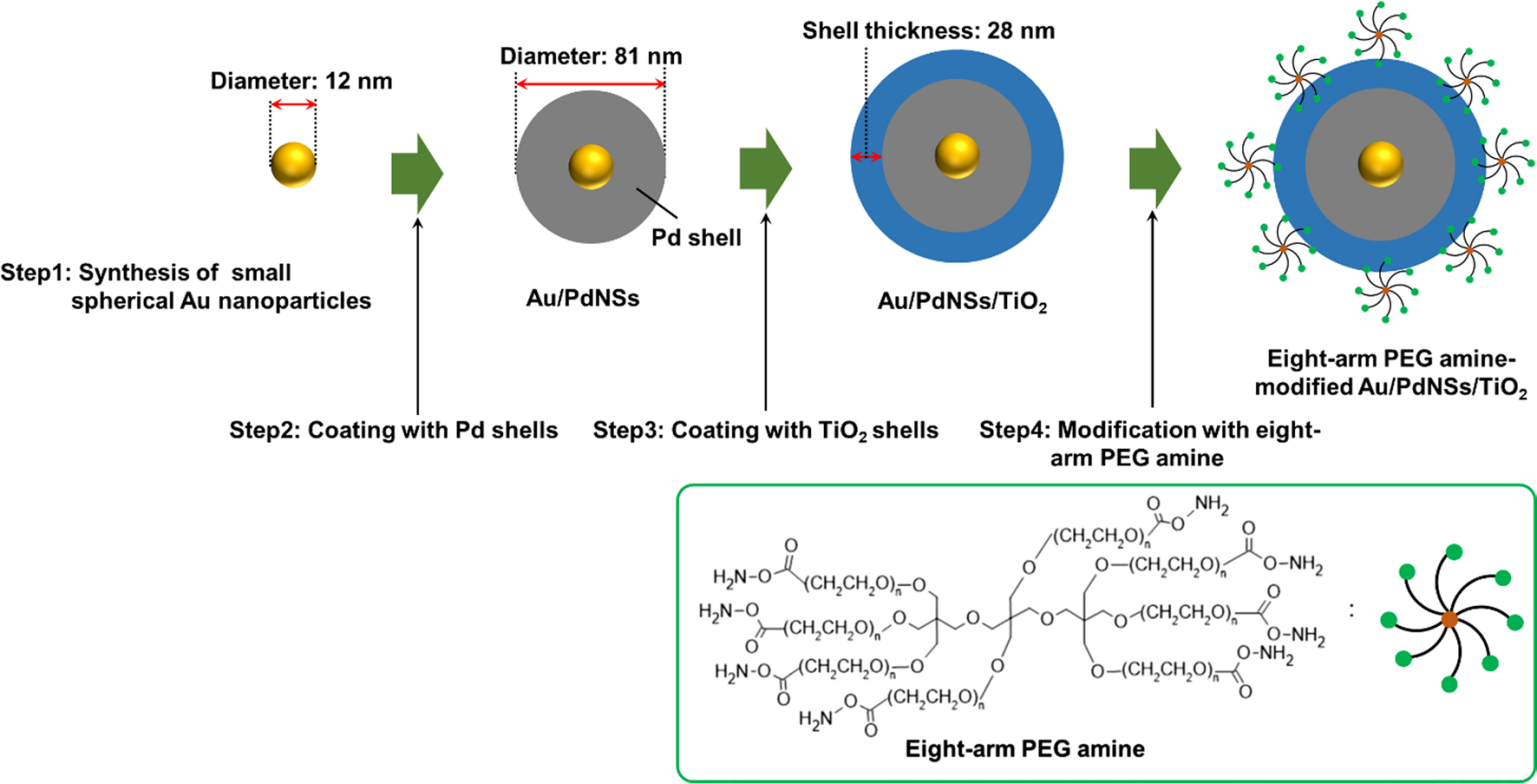
Scheme for preparation of Au/PdNSs/TiO_2_ and their surface modification

Small spherical Au nanoparticles were synthesized using a citric acid reduction method [31]. After an aqueous solution (1 wt%, 4 mL) of citric acid was added to an aqueous solution (0.01 wt%, 100 mL) of HAuCl_4_·4H_2_O, which was refluxed for 30 min in advance, the mixed solution was further refluxed for 1 h, resulting in the formation of spherical Au nanoparticles.

The Au/PdNSs were synthesized as follows [28, 32]. An aqueous solution of hydrogen tetrachloropalladate (II) (H_2_Pd_4_Cl_4_, 1 mM, 100 mL) was prepared by dissolving PdCl_2_ (17.7 mg) in an aqueous solution of hydrochloric acid (1 mM, 100 mL). Next, the colloidal aqueous solution of spherical Au nanoparticles (2.5 mL) was added into the aqueous solution of hydrogen tetrachloropalladate (II) (100 mL). Then, an aqueous solution (20 mL) containing of ascorbic acid (60 mM) and the citrate (0.1 wt.%) was slowly added dropwise into the mixed solution under stirring in an ice bath. After the addition was finished, the mixed solution was further stirred for 30 min in an ice bath, resulting in the formation of Au/PdNSs. The Au/PdNSs were purified by the centrifugation (12,000 rpm, 10 min), followed by redispersing in an aqueous solution of citric acid (0.1 wt.%, 25 mL).

### Coating Au/PdNSs with TiO_2_ shell (Au/PdNSs/TiO_2_, Step 3 in Scheme 1).

The coating of Au/PdNSs with TiO_2_ shell (Au/PdNSs/TiO_2_) was performed using a modified version of the previous report [33]. First, an aqueous solution of hydroxypropyl cellulose (0.5 wt.%, 360 μL) was added into the colloidal aqueous solution of Au/PdNSs (9 mL), followed by stirring the solution for 1 h. Next, after 2-propanol (36 mL) and ammonium aqueous solution (28%, 1 mL) were added to the mixed colloidal solution, a 2-propanol solution of TDAA (10 mM, 225 μL) was slowly added dropwise to the solution every hour three times under stirring, followed by stirring for 20 h, resulting in the formation of Au/PdNSs/TiO_2_. The obtained Au/PdNSs/TiO_2_ was purified by the centrifugation (12,000 rpm, 10 min) once, followed by redispersing in ethanol (10 mL). Finally, after the colloidal solution was diluted 10 times by ethanol, the solution was refluxed at 100 °C for 5 h.

### Modification of Eight-Arm PEG Amine on Au/PdNSs/TiO_2_ (Step 4 in Scheme 1)

The eight-arm PEG amine was modified onto Au/PdNSs/TiO_2_ by adding an ethanol solution of eight-arm PEG amine (10 mg/mL, 1 mL) into the colloidal ethanol solution of Au/PdNSs/TiO_2_ (1 mL), followed by stirring for 1 h. The colloidal solution was purified by the centrifugation (12,000 rpm, 10 min), followed by redispersing in Milli-Q water (1 mL).

### Measurements

The extinction spectra of resultant colloidal solution were measured using a V-770 spectrophotometer (JASCO). For the evaluation of the photothermal conversion properties of Au/PdNSs/TiO_2_ modified with 8-arm PEG amine, the temperature of the colloidal aqueous solution (2 mL) was measured using a thermocouple (OMEGA HW206) inserted in the solution under gentle stirring in a standard 1 cm quartz cell. A continuous wave (CW) laser with 808 nm (1.8 W) was applied to the colloidal solution. The distance between the laser source and surface of the cell was set to be 10 cm. Time variation of the temperature under the laser irradiation was measured up to arbitrary period, whereof the temperature of the colloidal solution reached a saturated state. Transmission electron microscopy (TEM) image was taken with a Hitachi HF-2000 with an acceleration voltage of 200 kV. The extinction, absorption, and scattering spectra of Au/PdNSs/TiO_2_ were calculated by analytical Mie theory using MatScat program on MATLAB [34, 35]. The Mie series was truncated by the Bohren–Huffman treatment [36], and the dielectric function of Pd and TiO_2_ was used from the previous reports [22, 37]. Surface analysis of Au/PdNSs/TiO_2_ was performed using XPS with an ESCA-3400 electron spectrometer (Shimadzu Co., Japan) at a base pressure of < 1.5 × 10^‑8^ Torr and a monochromatized Mg Kα (1253.6 eV) X-ray source.

### Evaluation of Cytotoxicity and Photothermal Therapeutic Effect of Au/PdNSs/TiO_2_

HeLa cell lines were cultured in D-MEM containing 1% penicillin/streptomycin and 10% FBS at 37 °C under 5% CO_2_. For the evaluation of cytotoxicity and photothermal therapeutic effect of Au/PdNSs/TiO_2_, HeLa cells (2.0 × 10^4^ cells/well, 700 μL) were seeded in four well plates and incubated for 24 h to allow the cells to attach to the bottom surface of the well. The colloidal aqueous solutions of Au/PdNSs/TiO_2_ modified with 8-arm PEG amine were centrifuged (10,000 rpm, 10 min) and redispersed in PBS (100 μL). After the addition of D-MEM (200 μL) into the PBS of Au/PdNSs/TiO_2_, the mixed solutions were added to the respective wells (concentrations: 100, 200, and 300 µg/mL). After incubating the cells with Au/PdNSs/TiO_2_ for 3 h and at 37 under 5% CO_2_, the medium containing the Au/PdNSs/TiO_2_ was removed. The D-MEM (700 μL) containing calcein AM (1 μM) and PI (0.5 μM), which selectively label the live and dead cells with green and red fluorescence, respectively, was added to the each well, followed by incubating for 20 min at 37 under 5% CO_2_, and then, the D-MEM was removed from the well. The cell observation was performed using a BX53 fluorescence microscope (Olympus). To evaluate the photothermal therapeutic effect, after incubating the cells with Au/PdNSs/TiO_2_ (concentrations: 100, 200, and 300 µg/mL) for 3 h at 37 under 5% CO_2_, the cells were exposed to a CW laser (808 nm, 1.8 W) for 5 min. After removing the medium from these systems, the cells, which were labeled with calcein AM and PI, were observed. The cell viability was determined by calculating the percentage of calcein-positive cells to the total number of cells.

## Results and Discussion

### Theoretical Optical Properties of Pd Nanosphere Coated with TiO_2_ Shell

**Fig. 1:**
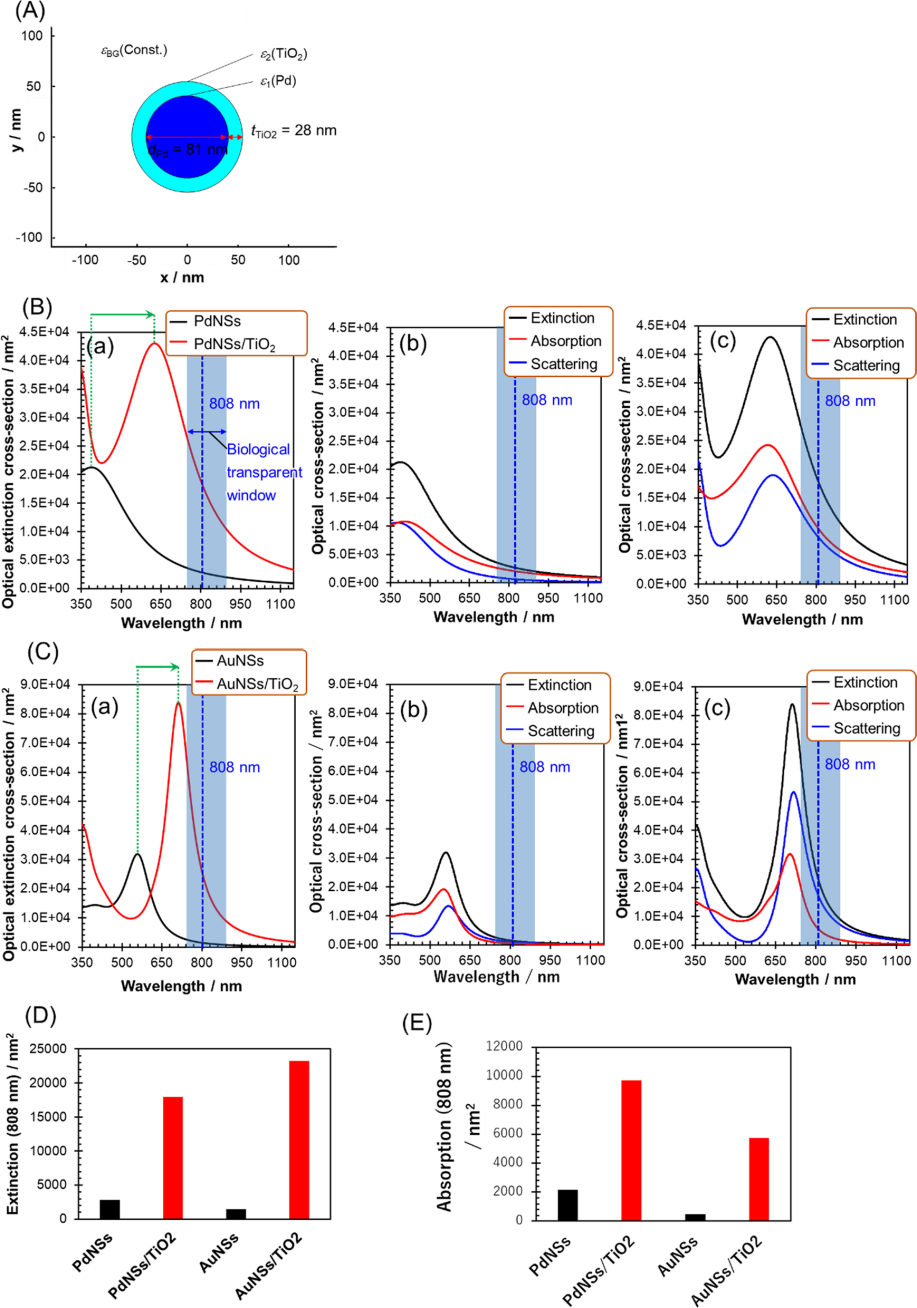
Optical properties of PdNS, PdNS/TiO_2_, AuNS, and AuNS/TiO_2_ calculated by the Mie theory. Dielectric function of Pd [22] and Au [54] was taken from the previous papers. Refractive index of TiO_2_ [37] was taken from the previous paper. (**A**) Geometrical model of PdNS/TiO_2_ for the calculation. (**B**) (a) Extinction spectra for PdNS and PdNS/TiO_2_ and extinction, absorption, and scattering spectra for (b) PdNS and (c) PdNS/TiO_2_. (**C**) (a) Extinction spectra for AuNS and AuNS/TiO_2_ and extinction, absorption, and scattering spectra for (b) AuNS and (c) AuNS/TiO_2_. (**D**) Extinction and (**E**) absorption cross sections of PdNS, PdNS/TiO_2_, AuNS, and AuNS/TiO_2_

We previously showed that the optical spectra of Au/PdNS core/shell particles can be approximated by those of homogeneous Pd nanospheres when the Au core is small enough. Therefore, we used Pd nanosphere as models for experimental Au/PdNS in the calculations. The optical characteristics of a Pd nanosphere (PdNS) which is coated with TiO_2_ shell (PdNS/TiO_2_) were compared with a bare PdNS and a spherical Au nanoparticle (AuNS) coated with TiO_2_ (AuNS/TiO_2_). The geometric models (Fig. 1A) of PdNS and PdNS/TiO_2_ were employed from the morphology of experimentally obtained Au/PdNS (average diameter: 81 nm) and Au/PdNS/TiO_2_ (shell thickness: approximately 28 nm, vide infra). The TiO_2_ thickness and PdNS core diameter were set to approximately 30 nm and 80 nm, respectively, to obtain the effective redshifting of LSP resonance of Pd and effective absorption cross section at 808 nm, which corresponds to the laser wavelength used in the *in vitro* cell tests (see Additional file 1: Figures S1 and S2). The properties of LSPR of metal nanoparticles can be described by the Mie theory, which gives analytical and exact solutions of the Maxwell equations for spherical nanoparticles with the multipole expansion of the electromagnetic fields [36]. In the extinction spectrum ((a) in Fig. 1B) of PdNS with a diameter of 81 nm simulated on the basis of the Mie theory, a broad extinction band attributed to the dipole mode of Pd LSPR was observed at 388 nm in the visible region. The band was extended to the biological transparent window including 808 nm, which corresponds to the laser wavelength used for in vitro photothermal therapeutic performance. The extinction cross section at 808 nm was 2.83 × 10^3^ nm^2^ (Fig. 1D). The coating with TiO_2_ with the thickness of 28 nm on PdNS redshifted the band by 237 nm, resulting in the maximum extinction wavelength at 625 nm ((a) in Fig. 1B). The large redshift is due to the inherently great refractive index sensitivity of Pd LSPR [28]; the refractive index of amorphous TiO_2_ (*n* = 2.5) is significantly higher than that (*n* = 1.333) of surrounding water. Note that the extinction cross section at 808 nm of Pd LSPR was increased by 6.35 times (18.0 × 10^3^ nm^2^) as shown in Fig. 1D. Next, the fraction of absorption component in the extinction at 808 nm should be considered because the deactivation process of LSP resonance can be divided into absorption (nonradiative decay), which directly contributes to the photothermal conversion, and scattering (radiative decay). The extinction, absorption, and scattering spectra for PdNS and PdNS/TiO_2_ are shown in (b) and (c) in Fig. 1B, respectively. For both nanoparticles, the absorption cross section is slightly larger than that of scattering cross section from the maximum extinction wavelengths to the longer wavelengths (PdNS: 76% and PdNS/TiO_2_: 54% for absorption/extinction ratio at 808 nm). The higher absorption/extinction ratios, compared to spherical Au nanoparticles (vide infra), are due to the effective coupling between the free- and bound-electron response of Pd. Consequently, the absorption cross section (9.73 × 10^3^ nm^2^) of PdNS/TiO_2_ was approximately 4.5 times higher than that (2.15 × 10^3^ nm^2^) of PdNS (Fig. 1E).

Next, theoretical calculations were also done for AuNS and AuNS/TiO_2_ with the same geometries to highlight the unique nature of the LSP resonance properties of Pd (PdNS and PdNS/TiO_2_). As shown in (a) in Fig. 1C, the maximum extinction wavelength of LSP resonance of AuNS was redshifted by 154 nm by coating with the TiO_2_ shell. The magnitude of the redshift was smaller than that for PdNS because the refractive index sensitivity of AuNS was smaller than that of PdNS [28]. The LSP resonance bands of both AuNS (558 nm) and AuNS/TiO_2_ (712 nm) exhibited their maximum at longer wavelengths than those of PdNS and PdNS/TiO_2_. However, the bands of Au nanospheres are significantly sharper than those of Pd nanospheres, and consequently, the absorption cross sections of Au nanospheres are smaller than those of Pd nanospheres at 808 nm (Fig. 1E). Particularly, the absorption cross section of AuNS/TiO_2_ was 41% lower than that of PdNS/TiO_2_.

### Morphological and Optical Characterization of Au/PdNSs/TiO_2_

**Fig. 2:**
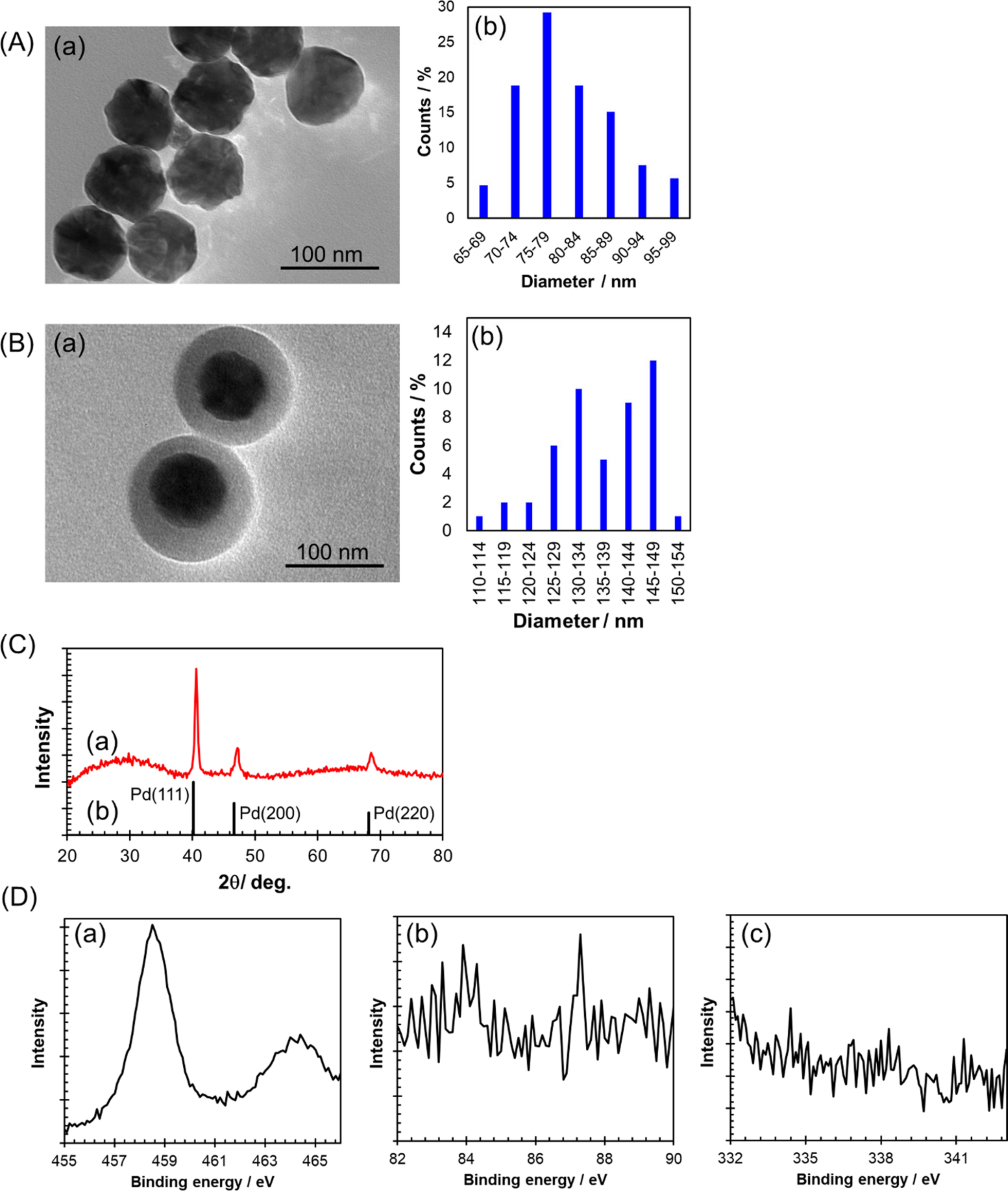
Morphological characterization of Au/PdNSs and Au/PdNSs/TiO_2_. (a) TEM image and (b) size distribution for (**A**) Au/PdNSs and (**B**) Au/PdNSs/TiO_2_. (**C**) (a) XRD patterns of Au/PdNSs/TiO_2_ and (b) JCPDS data of palladium. (**D**) XPS spectra of Au/PdNSs/TiO_2_ mounted on a glass plate. Narrow spectra for (a) Ti2p, (b) Au4f, and (c) Pd3d. The binding energies were corrected using the C1s level at 284.8 eV as an internal standard

We previously shown that the LSP resonance wavelength of PdNS (dipole mode) is longer for larger particles and was in near ultraviolet to visible region at with diameters above 40 nm [28]. On the other hand, the nanoparticles of which the diameter is too large (> 100 nm) can lead to the reduced dispersion stability in an aqueous phase. Therefore, we synthesized PdNSs with a diameter of approximately 80 nm. These Au/PdNSs were synthesized by seed-mediated growth method using small spherical Au nanoparticles (average diameter: 13.7 ± 1.9 nm, Additional file 1: Figure S3) as a core because the PdNSs with precisely controlled diameters have been successfully prepared by this method. Figure 2A shows the TEM image of Au/PdNSs and the size distribution calculated from the multiple similar TEM images. It was confirmed that Au/PdNSs formed a spherical shape with rugged surface. The highest abundance of nanoparticles was in the range of 75–79 nm, and the average diameter was 81.0 ± 7.5 nm. The Au/PdNSs were coated with the shell of TiO_2_ (Au/PdNSs/TiO_2_) as a high refractive index semiconductor, because TiO_2_ nanomaterials are well known to show high biocompatibility [38, 39]. The TEM image confirmed that the Au/PdNSs were coated by the TiO_2_ shell ((a) in Fig. 2B). The size distribution of Au/PdNSs/TiO_2_ ((b) in Fig. 2B) was relatively broad over 125–149 nm, and the average diameter was 136.9 ± 9.6 nm. Therefore, the thickness of TiO_2_ shell was estimated to be approximately 28 nm. Any peaks attributed to the crystalline TiO_2_ were not observed in the XRD spectrum of Au/PdNSs/TiO_2_ (Fig. 2C), which indicate that the coating TiO_2_ is amorphous, while the diffraction peaks of crystalline palladium, which matched with the data of Joint Committee on Powder Diffraction Standards (JCPDS), were observed [33]. Next, the XPS spectra for Au/PdNSs/TiO_2_ mounted on a glass plate were measured to further evaluate the TiO_2_ shell on Au/PdNSs (Fig. 2D). In the narrow spectrum of Ti2p, doublet peaks were observed at 458.5 eV and 464.3 eV, which were ascribed to Ti2p3/2 and Ti2p1/2 of Ti(IV), respectively. In addition, any peaks of Au4f and Pd3d were not observed. These results indicate that the surface of Au/PdNSs was fully coated by TiO_2_ shell.

**Fig. 3:**
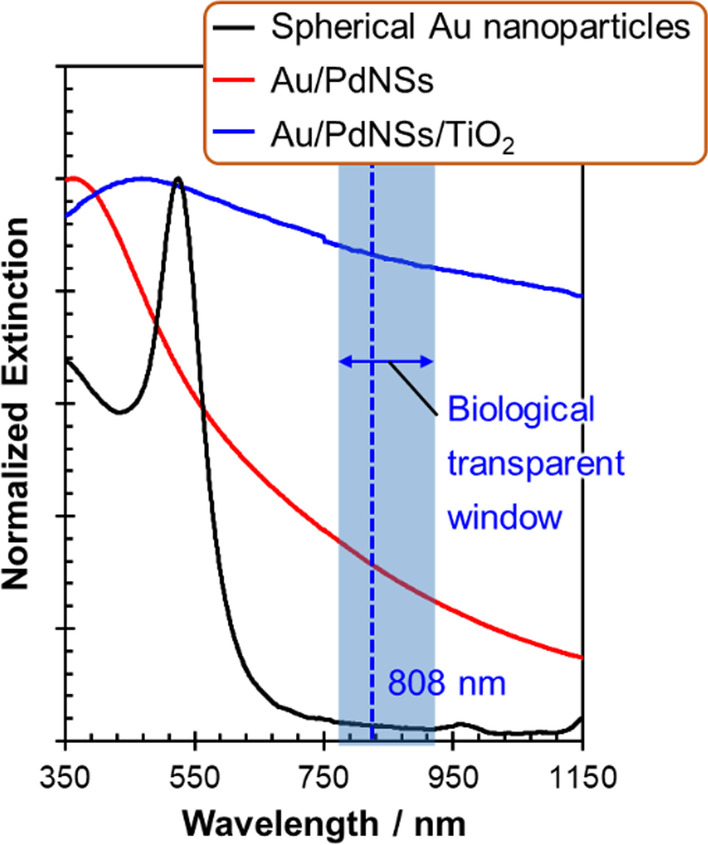
Extinction spectra of colloidal aqueous solutions of spherical Au nanoparticles, Au/PdNSs and Au/PdNSs/TiO_2_

The optical spectra of the synthesized Au/PdNSs/TiO_2_ are shown in Fig. 3. The extinction spectrum of the colloidal aqueous solution of small Au nanoparticles (core only) showed an LSP resonance peak at 525 nm. On the other hand, in the colloidal aqueous solution of Au/PdNSs, no Au-derived band was observed; instead, a broad extinction band with the maximum at 360 nm derived from the Pd LSP resonance was observed. This is consistent with our previous results that the LSP resonance of Au nanoparticle cores was completely shielded by the thick Pd shell (Additional file 1: Figure S4) [28]. Therefore, our synthesized Au/PdNSs can be optically treated as a PdNSs. Next, the maximum extinction wavelength of the LSP resonance of Au/PdNSs/TiO_2_ was 468 nm, which was redshifted by 108 nm compared to that of Au/PdNSs. This large redshift, which is attributed to the increase in the surrounding refractive index by coating with TiO_2_ shell, was qualitatively consistent with the calculated results as shown in (a) of Fig. 1(B), although the magnitude of the redshift was somewhat smaller than the calculated value (237 nm) and the obtained resonance band was broader. The overestimation of the redshift by calculation for the TiO_2_-coated nanoparticles may be derived from the difference between the calculated (388 nm) and observed (360 nm) LSPR peak values for the Pd cores, which may be further amplified by incorporating the high refractive index shell because the refractive index sensitivity increases with wavelength [28]. On the other hand, the broader observed LSPR band may be attributed to the wide distribution of experimental TiO_2_ shell thickness (shown in (b) in Fig. 2B). In any case, the redshifted and broad LSPR extending to the near-IR region implied that Au/PdNS/TiO_2_ could be a useful photothermal therapy agent, particularly because of the high extinction intensity at 808 nm.

### Photothermal Conversion Properties of Au/PdNSs/TiO_2_

**Fig. 4:**
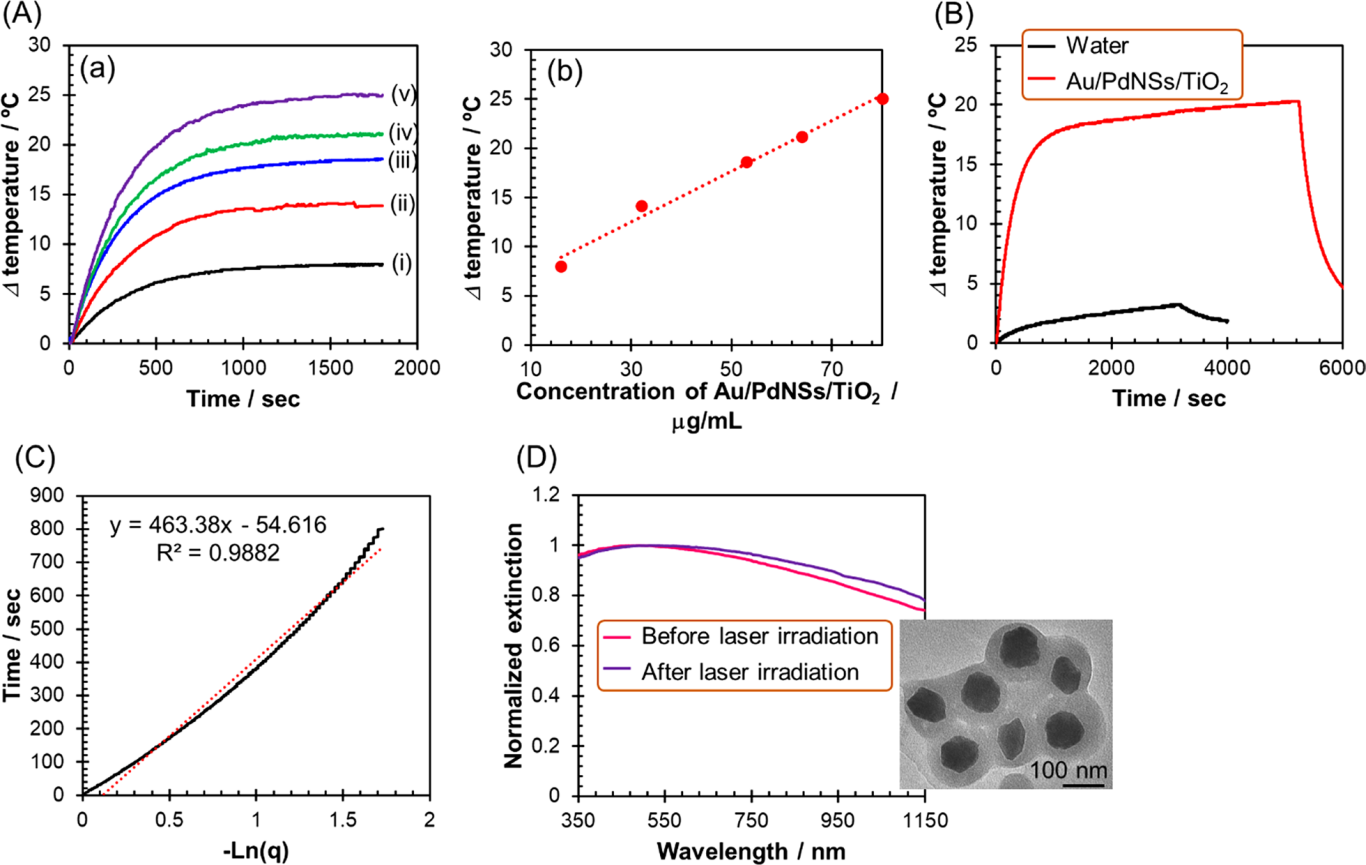
Photothermal conversion properties and stability against laser irradiation of Au/PdNSs/TiO_2_. (**A**) (a) The time-dependent temperature profiles of colloidal aqueous solutions of Au/PdNSs/TiO_2_ with concentrations of (1) 16, (2) 32, (3) 53, (4) 64, and (5) 80 μg/mL with the laser irradiation (808 nm, 1.8 W, irradiation period: 30 min). (b) Plots of maximum temperature rise against the concentration of Au/PdNSs/TiO_2_. (**B**) Temperature profiles of colloidal aqueous solution of Au/PdNSs/TiO_2_ and water as a reference under laser irradiation with 808 nm. (**C**) Time constant for heat transfer from the system was calculated by applying the linear time data from the cooling period *versus* negative natural logarithm of driving force temperature, which is obtained from (**B**). (**D**) Extinction spectra for Au/PdNSs/TiO_2_ before and after laser irradiation for ca. 90 min (inset: TEM image of Au/PdNSs/TiO_2_ after laser irradiation)

To investigate the photothermal conversion properties of Au/PdNSs/TiO_2_, the temperature changes induced by irradiation of 808 nm laser (1.8 W, 30 min) to the colloidal aqueous solutions with concentrations of 16, 32, 53, 64, and 80 μg/mL were recorded (Fig. 4A). As shown in (b) in Fig. 4A, the maximum temperature rise was linearly increased with increasing concentration of Au/PdNSs/TiO_2_, indicating that the temperature rise with the laser irradiation was caused by the photothermal conversion phenomenon of Au/PdNSs/TiO_2_. Also, to obtain the photothermal conversion efficiency (*η*), the laser irradiation to the colloidal aqueous solution with a concentration of 53 μg/mL (extinction intensity at 808 nm: 0.33) was continued until the temperature rise was saturated (Fig. 4B). The temperature rise was as much as 20.3, while pure water showed only a 2.8  increase under the same irradiation conditions. The *η* were calculated using Eq. 1 according to the method developed in previous reports [40, 41]

1 $$\eta = \frac{{{\text{hS}}(T_{\max } - T_{surr} ) - Q{}_{{{\text{dis}}}}}}{{I(1 - 10^{{ - {\text{A}}}} )}}$$

where *h* and S are the heat transfer coefficient and the surface area of the 1 cm quartz cell as a container. The *T*_max_ and *T*_surr_ are the maximum steady-state temperature and the temperature of the surrounding environment, respectively. The *Q*_dis_ represents the heat generated by pure water under the irradiation of 808 nm laser as shown in Fig. 4A. The lumped quantity *hS* is estimated using Eq. 2.

2 $$\tau_{s} = \frac{{\sum\limits_{i} {m_{i} C_{p,i} } }}{{{\text{hS}}}}$$

where *τ*_s_, *m*_i_, and *C*_pi_ are the system time constant, mass of water (*m*_H2O_) and quartz cell (*m*_cell_), and heat capacities of water (*C*_p,H2O_: 4.2 J/g) and quartz cell (*C*_p,cell_: 0.839 J/g) [42]. As presented in Fig. 4B, the *τ*_s_ value is estimated by fitting the cooling temperature profiles to Eqs. 3 and 4.

3 $$t = - \tau_{{\text{s}}} \ln \theta$$

4 $$\theta = \frac{{T - T_{{{\text{surr}}}} }}{{T_{\max } - T_{{{\text{surr}}}} }}$$

where *t* and *θ* are the cooling time and dimensionless driving force temperature, respectively. The *T* is the temperature of the system at time *t*. Consequently, the *η* was calculated to be 50%. Although the value was somewhat lower than the ratio of absorption in the extinction components (54%), this may be due to an increase in the scattering component owing to the rugged surface of Au/PdNSs ((a) in Fig. 2A). However, the efficiency is among the best values reported for some typical photothermal conversion materials consisting of anisotropic Au nanoparticles (Au nanorods: 21–63% [43–49], Au nanoshells: 13–33% [43, 44, 46, 49], Au nanostars: 28–31% [49, 50]) calculated based on Eq. (1), which suggests that Au/PdNSs/TiO_2_ can function as a superior photothermal therapy agent. Further, it was found that Au/PdNSs/TiO_2_ as well as Au/PdNSs is photothermally stable against the laser irradiation (1.8 W at 808 nm, 90 min), which was demonstrated by only a minor difference in the extinction spectra before and after the laser irradiation as shown in Fig. 4D and Additional file 1: Figure S5. Furthermore, the TEM images of Au/PdNSs (inset in Additional file 1: Figure S5) and Au/PdNSs/TiO_2_ (inset in Fig. 4D), which were taken from their colloidal solutions after the laser irradiation, showed that the original morphology was intact (Fig. 2B). These results indicate that our core–shell nanoparticles are stable against laser irradiation. It was reported that some anisotropic Au and Ag nanoparticles such as Au nanorods, Au nanostars, and Ag nanoprisms have low photothermal (and thermal) stability and easily melt from their apexes (protrusions) by laser irradiation or heat application [51, 52]. Therefore, the high photothermal stability for both of the present nanoparticles may be attributed to their isotropic morphology without any protrusions.

### In Vitro Evaluation of Biocompatibility and Photothermal Therapeutic Ability of Au/PdNSs/TiO_2_

The photothermal therapeutic ability of Au/PdNSs/TiO_2_ under the irradiation of 808 nm laser was investigated in vitro. For the application of Au/PdNSs/TiO_2_, the eight-arm PEG amine was modified on Au/PdNSs/TiO_2_ for efficient uptake into the cells and improved dispersion stability in PBS [53]. First, the cytotoxicity of Au/PdNSs/TiO_2_ was investigated under dark conditions using HeLa cells. The cytotoxicity was evaluated by co-staining the live and dead cells with calcein AM and propidium iodide (PI), respectively. The cell viability was calculated using Eq. 5:

5 $${\text{Cell viability}} = \frac{{\text{Number of cells stained with calcein AM}}}{{\text{Total number of cells stained with both calcein AM and PI}}}$$

**Fig. 5:**
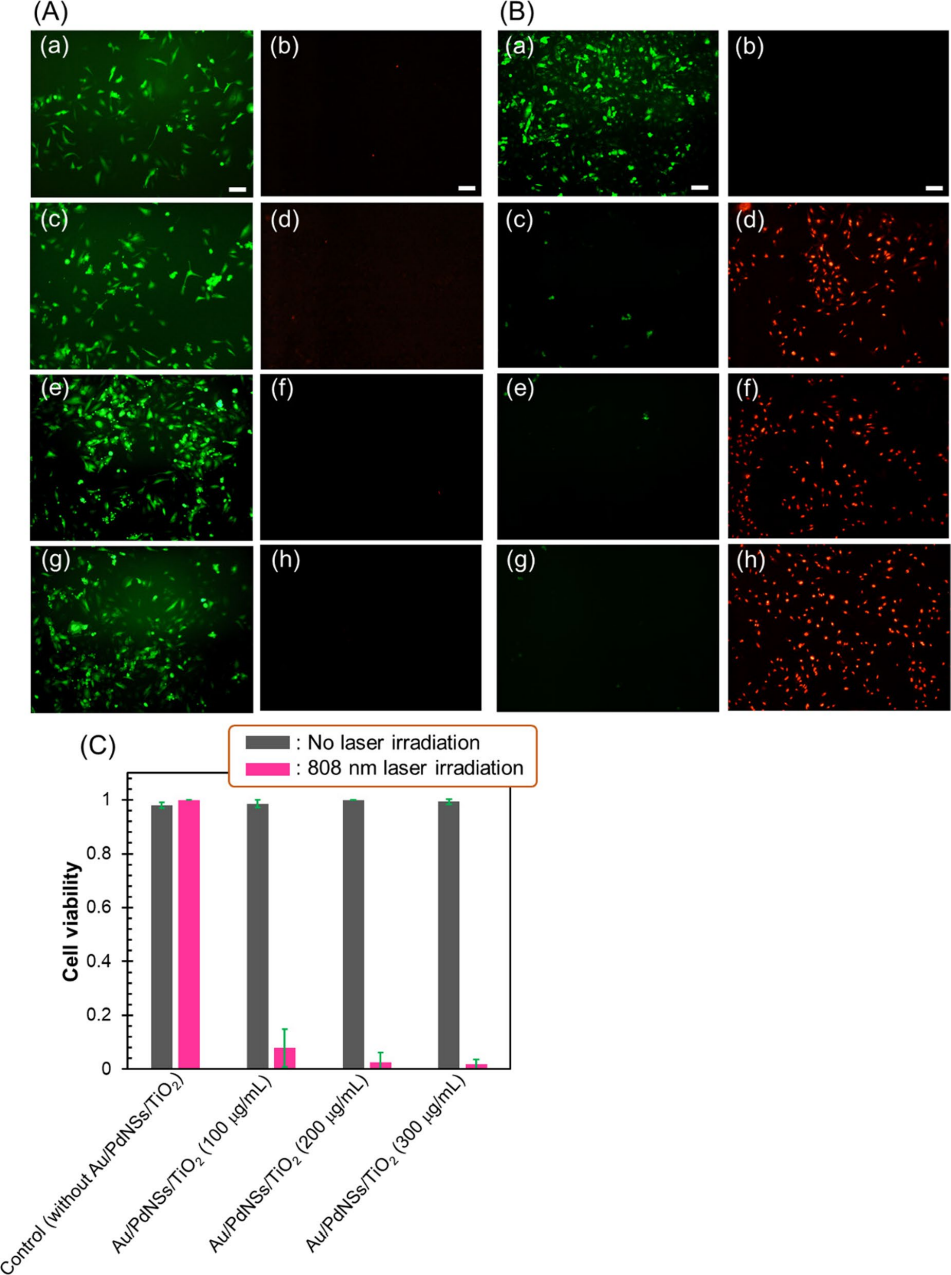
Cell viability after incubating HeLa cells with Au/PdNSs/TiO_2_. (**A**) Fluorescence images of calcein AM and PI-co-stained HeLa cells, which were incubated (a) without and with the concentrations of (b) 100, (c) 200, and (c) 300 μg/mL Au/PdNSs/TiO_2_. (**B**) Fluorescence images of calcein AM and PI-co-stained HeLa cells, which were incubated (a) without and with the concentrations of (b) 100, (c) 200, and (c) 300 μg/mL Au/PdNSs/TiO_2_, after laser irradiation with 808 nm (1.5 W). The scale bars of all images equal to 100 µm. (**C**) Quantitative cell viability obtained from respective conditions of (**A**) and (**B**)

Appropriate application of Eq. 5 requires that the total number of cells is more or less the same in each well. That this condition was met is shown by the counting data (concentration of Au/PdNSs/TiO_2_, total number of cells): 0 μg/mL, 95 ± 8 mm^−2^; 100 μg/mL, 99 ± 22 mm^−2^; 200 μg/mL, 118 ± 26 mm^−2^; and 300 μg/mL, 114 ± 8 mm^−2^. As shown in Fig. 5A and C, the viability of cells incubated with Au/PdNSs/TiO_2_ for all concentrations was high (> 98%), which was similar to that (98.1 ± 1.1%) obtained without the nanoparticles. Next, with irradiation with 808 nm laser, the cellular viability was decreased with the increasing concentration of Au/PdNSs/TiO_2_ with the lowest viability being 1.8% at the highest concentration (300 μg/mL) as shown in Fig. 5B and C. Since the laser irradiation to the cells without Au/PdNSs/TiO_2_ did not reduce the cell viability (100. 0 ± 0.0%), the large decrease in the cellular viability was attributed to the photothermal therapeutic effect of Au/PdNSs/TiO_2_, demonstrating that the Au/PdNSs/TiO_2_ functioned as a photothermal therapy agent.

## Conclusion

In this study, the authors focused on the control technique of the resonance wavelength of PdNSs utilizing a high refractive index sensitivity of Pd LSPR. It was theoretically and experimentally demonstrated that the LSPR was redshifted from ultraviolet to visible region by coating with TiO_2_ shell. In addition, it was theoretically found that the absorption cross section at 808 nm, which governs the photothermal conversion ability, was largely improved by the redshifting. It was demonstrated from the experimental *in vitro* cell tests that the death of HeLa cells was induced by exciting the LSPR of PdNSs associated with the laser (808 nm) irradiation. These results suggest that hybridization of PdNSs with high refractive index TiO_2_ was important for improving the photothermal therapeutic effect. Furthermore, we surmise that combining with materials with even higher refractive indices could further improve the photothermal therapeutic effect of PdNSs. In addition, in vivo experiments are currently underway to assess the practicality of these unique photothermal therapeutic nanomaterials. Currently, we are proceeding with research according to the strategy.

## Supplementary Information

**Additional file1:** **Fig. S1**. Optical properties of PdNS/TiO_2_ calculated by the Mie theory. **Fig. S2**. Optical properties of Au/PdNS/TiO_2_ calculated by the Mie theory. **Fig. S3**. TEM image of Au nanospheres. **Fig. S4**. Extinction, scattering, and absorption spectra of PdNS/TiO_2_ and Au/PdNS/TiO_2_ nanoparticles calculated by Mie theory. **Fig. S5**. Extinction spectra and TEM image for Au/PdNSs before and after the laser irradiation

## Data Availability

The datasets used or analyzed during the current study are available from the corresponding author on reasonable request.

## Abbreviations

LSPR: Localized surface plasmon resonance
PdNS: Palladium nanospheres
Au/PdNSs: Au(core)/Pd(shell) nanospheres
PdNS/TiO_2_: Palladium nanospheres coated with TiO_2_ shell
Au/PdNSs/TiO_2_: Au(core)/Pd(shell) nanospheres coated with TiO_2_ shell
Au: Gold
Pd: Palladium
