# Supplementary material for: Modulation Technique of Localized Surface Plasmon Resonance of Palladium Nanospheres by Coating with Titanium Dioxide Shell for Application to Photothermal Therapy Agent
Source: Nanoscale Res Lett. 2022 Jun 23;17:60. doi: 10.1186/s11671-022-03697-1 (PMC9226246; doi:10.1186/s11671-022-03697-1)
Supplement: Supplementary file 1 — Additional file1: Fig. S1. Optical properties of PdNS/TiO2 calculated by the Mie theory. Fig. S2. Optical properties of Au/PdNS/TiO2 calculated by the Mie theory. Fig. S3. TEM image of Au nanospheres. Fig. S4. Extinction, scattering, and absorption spectra of PdNS/TiO2 and Au/PdNS/TiO2 nanoparticles calculated by Mie theory. Fig. S5. Extinction spectra and TEM image for Au/PdNSs before and after the laser irradiation [file 11671_2022_3697_MOESM1_ESM.docx]

*Supporting Information for*

Modulation Technique of Localized Surface Plasmon Resonance of Palladium Nanospheres by Coating with Titanium Dioxide Shell for Application to Photothermal Therapy Agent

Yutaro Hayakawa,^1^ Masato Furuya,^1^ Hironobu Tahara,^2^ Yasuhiro Kosuge,^3^ Tsuyoshi Kimura,^4^ Kosuke Sugawa,^1*^ Joe Otsuki^1^

^1^Department of Materials and Applied Chemistry, College of Science and Technology, Nihon University, Chiyoda, Tokyo 101-8308, Japan

^2^Graduate School of Engineering, Nagasaki University, Bunkyo, Nagasaki, 852-8521, Japan

^3^Laboratory of Pharmacology, School of Pharmacy, Nihon University, 7-7-1 Narashinodai, Chiba, Funabashi 274-8555, Japan

^4^Institute of Biomaterials and Bioengineering, Tokyo Medical and Dental University, Chiyoda, Tokyo, 101-0062, Japan


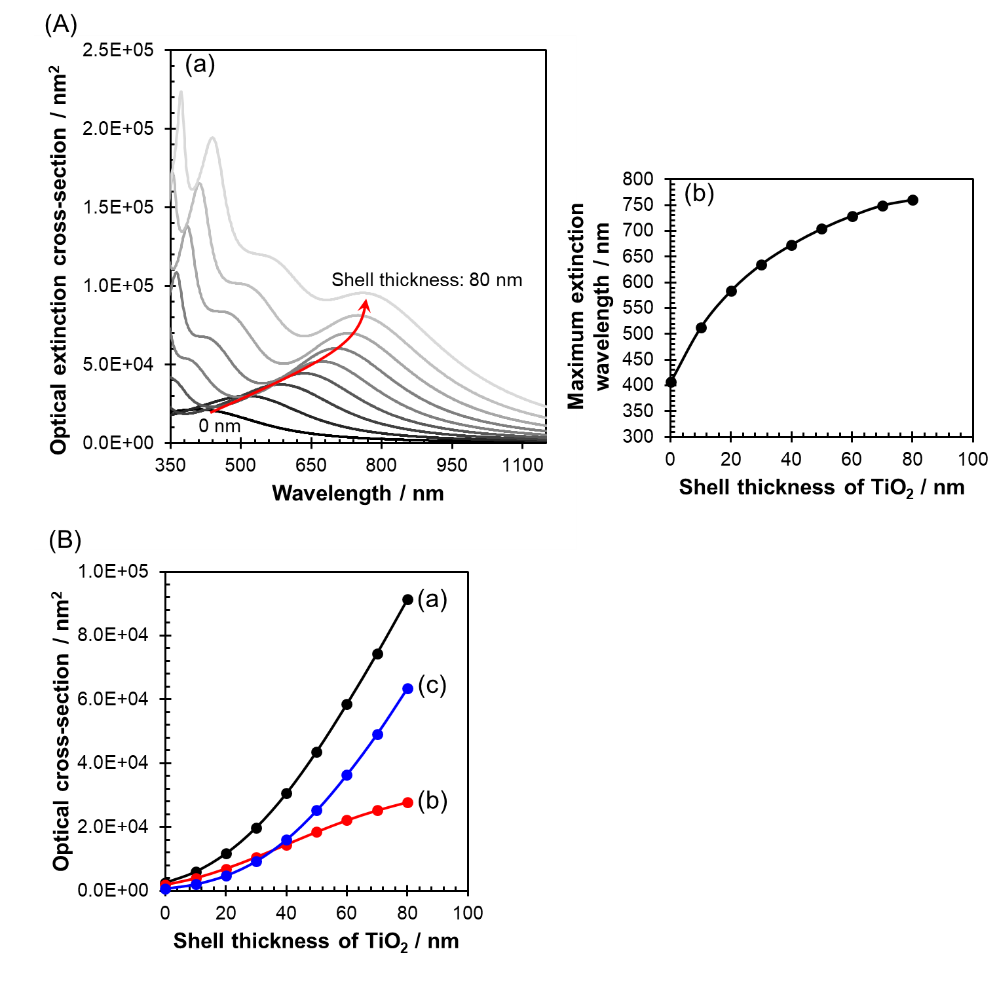


Figure S1. Optical properties of PdNS/TiO_2_ calculated by the Mie theory. (A) (a) Optical extinction spectra of PdNS/TiO_2_ and (b) plots of the maximum extinction wavelength of dipole mode of Pd LSP resonance of PdNS with varied TiO_2_ shell thickness (0-80 nm). (B) Optical cross-section of (a) extinction, (b) absorption, (c) scattering at 808 nm for PdNS/TiO_2_ with varied TiO_2_ (0-80 nm).


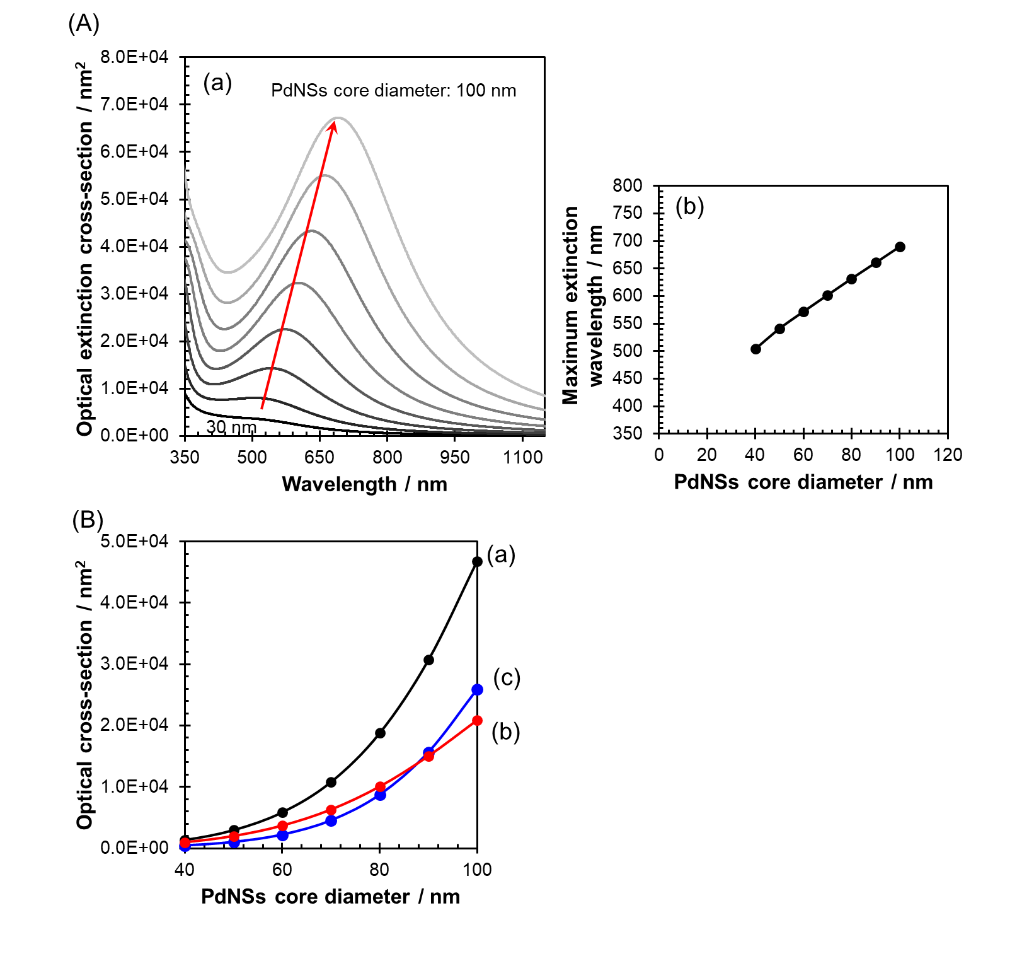


Figure S2. Optical properties of Au/PdNS/TiO_2_ calculated by the Mie theory. The Au nanoparticle diameter and TiO_2_ shell thickness are fixed at 14 and 30 nm, respectively. (A) (a) Optical extinction spectra of Au/PdNS/TiO_2_ and (b) plots of the maximum extinction wavelength with varied PdNS core diameter (30-100 nm). (B) Optical cross-section of (a) extinction, (b) absorption, (c) scattering at 808 nm for Au/PdNS/TiO_2_ with varied PdNS core diameter (40-100 nm).

As shown in Figure S1(A), the LSP resonance wavelength is red-shifted with increasing the thickness of TiO_2_ shell but the magnitude of shift becomes smaller above 30 nm. This is because that the refractive index sensitivity of LSP resonance becomes smaller at the position more away from the PdNS surface. In addition, the absorption cross-section of PdNS/TiO_2_, which is relevant to the photothermal conversion, follows a similar pattern (Figure S1(B)). When the thickness of TiO_2_ shell is greater than 40 nm, the scattering cross-section exceeds the absorption cross-section at 808 nm, reducing the photothermal conversion performance. Also, when the thickness of TiO_2_ shell is fixed at 30 nm, the maximum extinction wavelength is red-shifted with increasing the diameter of PdNS core (Figure S2(A)). However, when the PdNS core diameter is greater than 90 nm, the scattering cross-section at 808 nm exceeds the absorption cross-section (Figure S2(B)), leading to a decrease in photothermal conversion performance.


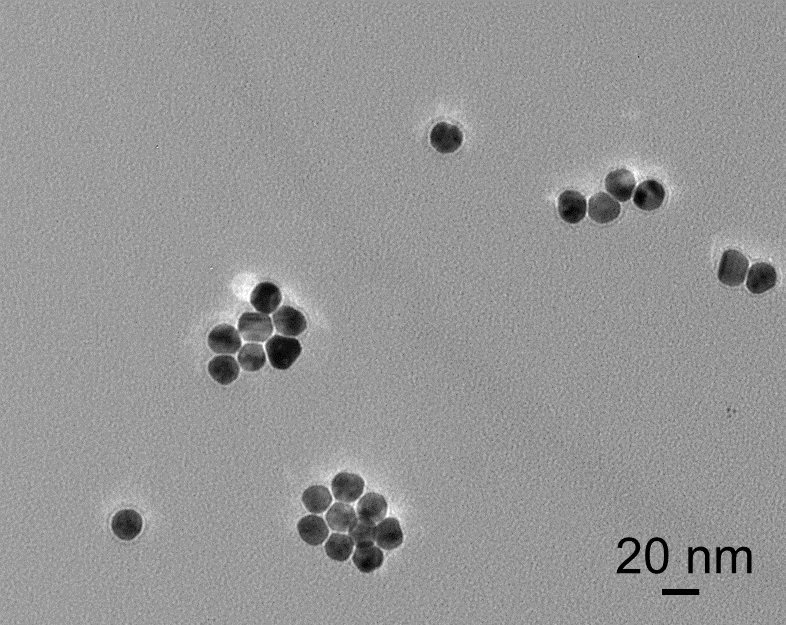


Figure S3. TEM image of spherical Au nanoparticles.

Figure S4. Extinction, scattering, and absorption spectra of PdNS/TiO_2_ and Au/PdNS/TiO_2_ nanoparticles calculated by Mie theory. Diameters of Au core and PdNS are 12 and 81 nm. Thickness of the TiO_2_ shell is 28 nm. Refractive index of the surroundings is 1.333. Multilayer effect was considered in Au/PdNS/TiO_2_ system by Mie theory and all dielectric functions of Au, Pd, and TiO_2_ were assumed to be invariant by the formation of core-shell structure.


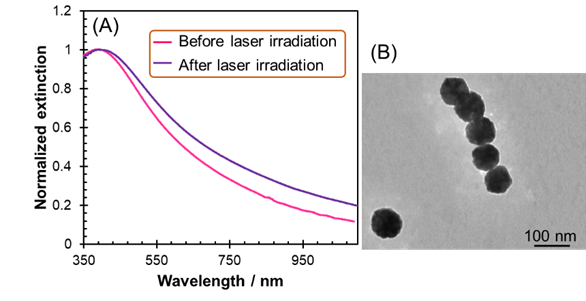


Figure S5. Extinction spectra for Au/PdNSs before and after the laser irradiation for ca. 90 min and the TEM image after laser irradiation.
